# Transcultural Adaptation, Validation, Psychometric Analysis, and Interpretation of the 22-Item Thai Senior Technology Acceptance Model for Mobile Health Apps: Cross-Sectional Study

**DOI:** 10.2196/60156

**Published:** 2025-03-11

**Authors:** Nida Buawangpong, Penprapa Siviroj, Kanokporn Pinyopornpanish, Wachiranun Sirikul

**Affiliations:** 1 Department of Family Medicine Faculty of Medicine Chiang Mai University Chiang Mai Thailand; 2 Department of Community Medicine Faculty of Medicine Chiang Mai University Chiang Mai Thailand; 3 Environmental and Occupational Medicine Excellence Center Faculty of Medicine Chiang Mai University Chiang Mai Thailand; 4 Center of Data Analytics and Knowledge Synthesis for Health Care Chiang Mai University Chiang Mai Thailand; 5 Department of Biomedical Informatics and Clinical Epidemiology Faculty of Medicine Chiang Mai University Chiang Mai Thailand

**Keywords:** STAM, senior technology acceptance model, validity, reliability, mHealth, older adult, technology acceptance, mobile health, app, transcultural adaptation, psychometric analysis, geriatrics, cross-sectional study, Thai, theory analysis, Cronbach α, McDonald ω, quality of life, well-being, social media, telehealth, health informatics, eHealth, mobile phone

## Abstract

**Background:**

The rapid advancement of technology has made mobile health (mHealth) a promising tool to mitigate health problems, particularly among older adults. Despite the numerous benefits of mHealth, assessing individual acceptance is required to address the specific needs of older people and promote their intention to use mHealth.

**Objective:**

This study aims to adapt and validate the senior technology acceptance model (STAM) questionnaire for assessing mHealth acceptance in the Thai context.

**Methods:**

In this cross-sectional study, we adapted the original, 38-item, English version of the STAM using a 10-point Likert scale for mHealth acceptability among the Thai population. We translated the mHealth STAM into Thai using forward and backward translation. A total of 15 older adults and experts completed the pilot questionnaire and were interviewed to assess its validity. The pilot items of the Thai mHealth STAM were then reworded and revised for better comprehension and cross-cultural compatibility. The construct validity of the Thai mHealth STAM was evaluated by a multidimensional approach, including exploratory and confirmatory factor analysis and nonparametric item response theory analysis. Discriminative indices consisting of sensitivity, specificity, and area under the receiver operating characteristic (AUROC) were used to determine appropriate banding and discriminant validity for the intention to use mHealth. Internal consistency was assessed using Cronbach α and McDonald ω coefficients.

**Results:**

Out of the 1100 participants with a mean age of 62.3 (SD 8.8) years, 360 (32.7%) were adults aged 45-59 years, and 740 (67.3%) were older adults aged 60 years and older. Of the 40-item pilot questionnaire, exploratory factor analysis identified 22 items with factor loadings >0.4 across 7 principal components, explaining 91.45% of the variance. Confirmatory factor analysis confirmed that 9-dimensional sets of 22 items had satisfactory fit indices (comparative fit index=0.976, Tucker-Lewis index=0.968, root mean square error of approximation=0.043, standardized root mean squared residual=0.044, and *R*^2^ for each item>0.30). The score banding D (low≤151, moderate 152-180, and high≥181) was preferred as the optimal 22-item Thai mHealth STAM cutoff score based on the highest sensitivity of 89% (95% CI 86.1%-91.5%) and AUROC of 72.4% (95% CI 70%-74.8%) for predicting the intention to use mHealth. The final Thai mHealth STAM, consisting of 22 items, exhibited remarkable internal consistency, as evidenced by a Cronbach α of 0.88 (95% CI 0.87-0.89) and a McDonald ω of 0.85 (95% CI 0.83-0.87). For all 22 items, the corrected item-total correlations ranged between 0.26 and 0.71.

**Conclusions:**

The 22-item Thai mHealth STAM demonstrated satisfactory psychometric properties in both validity and reliability. The questionnaire has the potential to serve as a practical questionnaire in assessing the acceptance and intention to use mHealth among pre-older and older adults.

## Introduction

As the global population ages, the integration of technology into the lives of older adults becomes increasingly crucial for enhancing their quality of life, independence, and well-being [1]. An emerging technology that promotes healthy aging is mobile health (mHealth). mHealth refers to medical and public health services facilitated by mobile devices [2]. It can provide individualized care plans for older adults to sustain functional ability and enhance quality of life [3]. Examples of mHealth innovations for older adults include supporting services for age-friendly health and facilitating the establishment of behavioral changes [3,4]. However, the adoption of technology, for example, mHealth, among older adults remains a complex and multifaceted issue, influenced by various factors such as individual perception and experience, ease of use, technological support, and sociocultural contexts [5,6]. To address this challenge, numerous theoretical frameworks have been proposed to understand and predict older adults’ acceptance of technology.

Assessing technology acceptance is essential for the successful implementation and use of mHealth technologies, as it directly influences user engagement, health outcomes, and health care delivery efficiency. Understanding acceptance helps developers create user-friendly applications [7,8], improves health outcomes through better adherence to interventions [9,10], and guides implementation strategies to address barriers effectively [11,12]. It also informs policy makers and administrators, enabling evidence-based decisions on mHealth investments [13,14]. Therefore, the lack of validated questionnaires for assessing technology acceptance could lead to a limited understanding of user needs and missed opportunities for improvement. Addressing this gap by developing and validating robust assessment is critical for maximizing the benefits of mHealth technologies and ensuring their effective adoption across diverse populations.

In the field of mHealth, various instruments and frameworks have been developed to assess adoption, intention to use, and acceptance. Established instruments like the Health Information Technology Usability Evaluation Scale (Health-ITUES) [15,16], System Usability Scale (SUS) [17,18], and mHealth App Usability Questionnaire (MAUQ) [19,20] provided insights focusing on user experiences and satisfaction. Broader frameworks include the unified theory of acceptance and use of technology (UTAUT) [21,22], which was extended to include additional factors relevant to mHealth, such as trust and perceived reliability, and was used in various studies to predict mHealth acceptance; the Fit between Individuals, Tasks, and Technology (FITT) Framework [23] is another, which was introduced to measure acceptance in clinical environments, emphasizing the alignment between user needs and technology capabilities. Despite their usability, these instruments and frameworks often lack specificity when addressing the unique needs of older adults.

The senior technology acceptance model (STAM) [24] stands out due to its tailored approach for older adults, which addresses their unique challenges and enhances the relevance of mHealth technologies for this population, making it more relevant than general models like the technology acceptance model (TAM) [25] or the UTAUT [26]. Furthermore, it emphasizes the role of social influence and support, which are critical for older adults who may rely on family and caregivers for technological adoption and addressing common health conditions in older adults, such as cognitive load and physical limitations.

The STAM was first proposed by Chen and Chan [24] in 2014 and has gained prominence for its focus on the unique needs and characteristics of older adults. This model was developed based on a study of 1012 older adults aged 55 years and older in Hong Kong, and it specifically targets older adults as its primary population of interest. The STAM integrates concepts from established technology acceptance frameworks, such as the TAM [25] and the UTAUT [26], tailored to address the specific considerations of older adults and provides a thorough framework for studying the factors that influence technological adoption in this age group. The study indicated 8 dimensions associated with technology acceptance in older adults, which included gerontechnology self-efficacy, gerontechnology anxiety, facilitating conditions, self-reported health conditions, cognitive ability, social relationships, attitude toward life and satisfaction, and physical functioning. Sociodemographic factors such as age, gender, education, and economic status are taken into account [24].

While the STAM has been used in different cultural contexts in other Asian countries, including Hong Kong [24] and South Korea [27], its applicability to the Thai population has not been validated. Thailand, like many other countries, is experiencing rapid population aging, emphasizing the urgency of understanding and promoting health technology acceptance among older adults [28]. However, cultural background, social norms, and technological infrastructures specific to Thailand may influence older adults’ perceptions and behaviors toward technology differently than in other contexts. Therefore, this study aimed to adapt, validate, and define the interpretation of the STAM questionnaire for evaluating the acceptance and intent to use mHealth in Thailand.

## Methods

### Study Design and Study Population

The cross-sectional study was conducted from August 2022 to July 2023 through a nationwide, web-based survey and a community survey. Eligible criteria for the study were Thai citizens aged 45 years and older on the date of the survey who could read and communicate in the Thai language and had no underlying conditions or diseases that limited their ability to complete the survey or use mHealth apps (eg, dementia, active psychological problems, or severe visual problems). The web-based survey was disseminated through an assortment of social media platforms, including the department websites, Facebook, Line, Twitter (rebranded as X in 2023), and Instagram. The information on community survey setting and recruitment is described in the section below. For the survey data collection, the respondents to both the web-based and community surveys used the Research Electronic Data Capture (REDCap; Vanderbilt University) survey platform to self-complete the questionnaires. REDCap [21,22] is a secure, web-based software platform designed to support data capture for research studies, providing (1) an intuitive interface for validated data capture, (2) audit trails for tracking data manipulation and export procedures, (3) automated export procedures for seamless data downloads to common statistical packages, and (4) procedures for data integration and interoperability with external sources. All study data were collected and managed using REDCap tools hosted at the Faculty of Medicine, Chiang Mai University. All respondents provided their informed consent, which was included in the screening questionnaire and study information sheet, before participating in this survey. The study excluded incomplete respondents or participants who spent less than 2 minutes or more than 60 minutes on the survey. This study was reported in accordance with COSMIN (Consensus-Based Standards for the Selection of Health Status Measurement Instruments)

reporting guidelines for studies on measurement properties of patient-reported outcome measures [29].

### Community Survey Setting and Recruitment

The community survey was distributed by the investigator team, consisting of medical students and health care personnel at primary care units from 10 subdistricts in Chiang Mai province. To identify eligible participants in the target area, officers from the subdistrict primary care units reviewed periodic health survey data for community-dwelling adults aged 45 years and older. Subsequently, patients’ information was verified with the health-promoting hospital databases to exclude individuals with health conditions that impeded survey participation or mHealth use as described above. The subdistrict primary health care teams invited eligible individuals to participate in the study through individual contact by community health care volunteers, community radio announcements, and posters displayed at primary care units.

### Ethical Considerations

The ethical consideration of the human subject research was approved by the Institutional Review Board of the Faculty of Medicine, Chiang Mai University (COM-2565-09079). All respondents provided their informed consent, as outlined in the screening questionnaire and study information page, before participating in this survey. For the web-based survey, respondents remained anonymous, and no identification data were recorded. In the case of the community survey, identification data of eligible participants were used solely for recruitment purposes within each target area and were not recorded in either the survey form or the study database. Participants received 100 Thai Baht (US $3) as compensation for answering the questionnaires.

### Translation and Adaptation of the Thai mHealth STAM

The original, English, 38-item STAM is a 10-point Likert scale consisting of 10 subscales and 38 items that capture the acceptance of general technology use for the older adult population. The total ranges from 38 to 380 points, with a higher score indicating greater acceptance of technology. The validity and reliability of 38-item STAM have been established on a satisfactory scale in 1012 older adults aged 55 years and older in Hong Kong [24]. The construct validity of the STAM was also evaluated with the confirmatory factor analysis (CFA) and revealed a satisfactory model fit with the proposed structure (comparative fit index [CFI]=0.938, root mean square error of approximation [RMSEA]=0.054, and standardized root mean square residual [SRMR]=0.075). The reliability of each subscale with Cronbach α coefficients ranged from 0.67 to 0.95.

Translation and adaptation of the Thai mHealth STAM was performed in accordance with the second edition of the International Test Commission (ITC) Guidelines for Translating and Adapting Tests [30]. In accordance with the ITC precondition guidelines, permission from the holder of the intellectual property rights relating to the 38-item STAM was obtained before performing any translation and adaptation of the STAM. The forward and backward translation with an expert reconciliation design was performed as recommended by the ITC test development guidelines. Before beginning the forward translation process, we decided to include a new subscale, perceived barriers, in the Thai STAM version due to the findings from the previous scoping review [31] on adopting mobile apps for health-related interventions among older adults. It revealed that barriers to adopting mHealth apps among older adults were the most common topics identified in the included studies. Insufficient technological skills, perceived lack of capability and time, concerns regarding personal data privacy, and trust in mHealth providers were the four items comprising the perceived barriers subscale. Following the translation protocol, the original, English, 38-item STAM was adapted to specify mHealth apps in all items and then forward translated into Thai by a professional translator to ensure accuracy for the target audience. The expert panel, which included a digital health expert (family physician and epidemiologist), 2 gerontology physicians, and a public health expert in community medicine, reviewed the forward translation of the Thai STAM questionnaire to ensure readability and transcultural adaptation. The backward translation was done by another professional translator into English. Then, the expert panel reconciled the backward translation version with the original STAM version. The investigator’s team resolved any discrepancies by reaching a final consensus through discussions with the expert panel. To ensure the face and content validity of the proposed questionnaire, a literature review, an expert review, and public interviews were incorporated into the adaptation of the Thai mHealth STAM. In total, 15 older adults participated in this phase to complete the pilot 40-item Thai STAM. Participants were subsequently interviewed to assess the following: overall questionnaire readability, clarity of instructions and items/response options, comprehension of the questionnaire, and other feedback on each item. Then, the pilot 40-item Thai STAM was reworded and revised as recommended on input from both participants and expert interviews. Finally, the pilot 40-item Thai mHealth STAM was given to a group of 40 older adults to verify its reliability and scale usability.

### Sample Size Estimation

The sample size was estimated based on three parameters, which are as follows: (1) a stable structure for an exploratory factor analysis (EFA) based on the rule of thumb, which is 10 cases per question; (2) expected CFI for a CFA based on the structural equation modeling; and (3) expected Cronbach α for the internal consistency of the questionnaire. For the first parameter, according to the rule of thumb, at least 440 respondents, accounting for 10% of the dropout rate, were required for an EFA. To achieve the expected CFI of 0.95 for a CFA, at least 459 respondents, accounting for 10% of the dropout rate, were required based on an average factor loading of 0.60 and an average factor correlation of .30 to ensure a .05 α (type I) error and power of 90% [32]. For testing overall reliability, at least 146 total respondents were required based on expected Cronbach α=0.80 (SD .05), a confidence level of 95%, and a dropout rate of 10% [33]. All sample size estimation was performed by the web-based sample size calculator [34]. Finally, the minimal required sample size for this study was 920, which was divided into 460 each for the EFA and CFA, respectively.

### Statistical Analysis

#### Descriptive Analysis

All statistical analyses were conducted using Stata (version 17.0; StataCorp). A *P* value below .05 indicated statistical significance. Categorical data were presented as frequency and percentage, while continuous data were described using mean (SD). Univariable analysis for comparison was performed as appropriate. The Thai mHealth STAM item scores were summarized with central estimations, measures of variability, floor and ceiling effect, skewness, and kurtosis tests. The overall psychometric properties of the Thai mHealth STAM were evaluated for validity and reliability as follows:

#### Dimensionality

To explore and reduce the dimensionality of the proposed questionnaire, an EFA was performed using a principal component analysis (PCA). The selection of PCA over common factor analysis was based on its ability to enhance parsimony and aid in the selection of factors for CFA [35]. Communalities were initially evaluated, and then orthogonal rotation with the varimax criteria and oblique rotation with promax criteria of the component was conducted. The Kaiser-Meyer-Olkin (KMO) measure and the Bartlett test of sphericity were conducted to verify the appropriateness of using factor analysis. A KMO value greater than 0.8 [36] and a Bartlett test with a *P* value less than .05 [37] are suggested for assessing sample adequacy and the suitability of the data for factor analysis, respectively. Eigenvalues greater than 1, the cumulative percentage of variance, and the scree plot with the number of factors that explained more than 5% of the variance were used to determine the number of factors to be retained [38,39]. A parallel study was conducted to validate the optimal threshold for the number of included factors [40]. Then, we used the following criteria to evaluate the adequacy of the EFA results. First, each should be saliently loaded with at least three items to ensure reliability and stability. In case a factor contains only 2 items, the expert panel consensus will be reached to ensure that the factor is meaningful based on the context and theoretical basis. Second, each item should load saliently on only 1 factor without complex or cross-loadings. Third, each factor should demonstrate internal consistency reliability ≥0.70. Fourth, all factors should be theoretically meaningful [35,41,42].

#### Construct Validity

For a CFA, structural equation modeling using a maximum likelihood estimation was performed to assure the factor structure based on the exploratory factor, as described previously. To determine the appropriateness of the proposed model, the specific fit indices were evaluated as follows: RMSEA<0.100, SRMR<0.100, CFI>0.900, and Tucker-Lewis Index (TLI)>0.900 [43-45]. To establish acceptance of the final structure of the final model, the coefficient of determination (*R*^2^) and item-scale correlation (standardized factor loading) should be at least 0.30 and 0.40, respectively. Finally, a nonparametric item response theory (IRT) analysis was done to confirm that the final Thai mHealth STAM had the unidimensional set for the relationship between the latent trait and the responses to the items [46]. The IRT analysis was assessed based on fundamental assumptions, including unidimensionality, local independence, and monotonicity. Loevinger H coefficients (*H^s^*) less than 0.3, between 0.3 and 0.4, and greater than 0.4, as determined by the item traces, correspond to poor, medium, and strong scalability properties, respectively. The monotonicity assumption criterion was determined by a critical value of less than 80.

#### Discriminant Validity

To determine the discriminant validity of the final questionnaire, the intention to use mHealth, as indicated in the external question, “If there are available mHealth applications for you, do you want to use them? (yes/no),” was used as the anchor-based question. The discriminative indices, including sensitivity, specificity, and area under the receiver operating characteristic (AUROC), were used with the intention of determining the appropriate cutoff scores. The 6 proposed bandings for the Thai mHealth STAM scores are categorized into low, moderate, and high acceptance based on score tertiles. Associations between these bandings and the intention to use mHealth are presented by adjusted odds ratios (aORs) with 95% CI from a multivariable logistic regression adjusted for potential confounders such as age, gender, education, income, and living alone.

#### Reliability

To estimate the correlation statistics for reliability, 95% CI using 1000 bootstrap resampling was presented alongside the reported correlation statistics. An internal consistency consisting of Cronbach α and McDonald ω coefficients was calculated for each item of the final questionnaire, as well as the entirety of the final questionnaire, to determine internal consistency, reliability, and the degree to which every item on a scale measures the same construct. The values of at least .70 indicated acceptable reliability of the questionnaire [47]. In addition, the item-total correlations and the corrected item-total correlations between .20 and .80 were considerably acceptable. A subgroup analysis of adults aged 45-59 years and adults aged 60 years and older was also performed, recognizing the importance of understanding the unique health needs and challenges faced by both current older populations and those who will age into this group in the future.

## Results

### Findings From the Translation and Adaptation of the Thai mHealth STAM

After reviewing the forward translation, the panel of experts decided to remove 2 items from the gerontechnology self-efficacy subscale, as they were redundant with the facilitating condition (FC) subscale (FC1 and FC2). Independent back-translation provided an additional check of the semantic equivalence of the translation. A total of 4 items, including PU2, PEOU2, P4, and P8, were modified based on the backward translation. For face and content validity, we conducted interviews with 15 older adults similar to the target population. Based on participants’ feedback, 4 items (FC1, FC2, C4, and P2) were slightly modified for clarity. In addition, 2 gerontology experts suggested rephrasing 2 items (A1 and A2) regarding attitude to aging and life satisfaction due to the sensitive wording. Finally, the 40-item Thai mHealth STAM in the pilot group of 40 older adults indicated acceptable internal consistency (Cronbach α =0.91). The details of the full 40 items (10 dimensions) of the Thai mHealth STAM are presented in Table S1 in Multimedia Appendix 1.

### Participant Characteristics

From the total of 1100 participants, the mean age was 62.3 (SD 8.8) years. The majority of participants were female (776/1100, 70.5%). Among the 1100 participants, 360 (32.7%) were adults aged 45-59 years, and 740 (67.3%) were older adults aged 60 years and older. Statistically significant differences in the characteristics between adults and older adults were observed in marital status (*P*=.003), education levels (*P*<.001), income (*P*<.001), underlying diseases (*P*<.001), and technology experience (*P*<.001). The characteristics of the participants of the study population are presented in Table 1. The derived data were randomly divided in a 1:1 ratio into 2 datasets in preparation for the EFA and CFA. The characteristics of the participants involved in the EFA and CFA are described in Table S2 in Multimedia Appendix 1.

**Table 1:** Participant characteristics of the study population.

Characteristics			Total (N=1100)		Adults (n=360)		Older adults (n=740)		*P* value	
Age (year), mean (SD)			62.3 (8.8)		52.4 (5.5)		67 (5.5)		<.001	
Male, n (%)			324 (29.5)		99 (27.5)		225 (30.4)		.32	
**Marital status, n (%)**										.003
	Single	96 (8.7)		43 (11.9)		53 (7.2)				
	Married	747 (67.9)		250 (69.4)		497 (67.2)				
	Separated, divorced, or widowed	257 (23.4)		67 (18.6)		190 (25.7)				
**Education levels, n (%)**										<.001
	No education	18 (1.6)		1 (0.3)		17 (2.3)				
	Primary school	725 (65.9)		160 (44.4)		565 (76.4)				
	Secondary school	97 (8.8)		50 (13.9)		47 (6.4)				
	High school and vocational training	162 (14.7)		99 (27.5)		63 (8.5)				
	Pre-university	11 (1)		6 (1.7)		5 (0.7)				
	Bachelor’s degree	79 (7.2)		41 (11.4)		38 (5.1)				
	Master’s degree	8 (0.7)		3 (0.8)		5 (0.7)				
**Income (THB^a^), n (%)**										
	<10,000	948 (86.2)		275 (76.4)		673 (90.9)		<.001		
	10,001-30,000	138 (12.5)		79 (21.9)		59 (8)				
	>30,001	14 (1.3)		6 (1.7)		8 (1.1)				
**Living status, n (%)**										.91
	Alone	108 (9.8)		34 (9.4)		74 (10)				
	With family	988 (89.8)		325 (90.3)		663 (89.6)				
	With others	4 (0.4)		1 (0.3)		3 (0.4)				
**Living area, n (%)**										.49
	Urban	220 (20)		78 (21.7)		142 (19.2)				
	Sub-urban	377 (34.3)		116 (32.2)		261 (35.3)				
	Rural	503 (45.7)		166 (46.1)		337 (45.5)				
Had any underlying disease, n (%)			726 (66)		189 (52.5)		537 (72.6)		<.001	
Hypertension, n (%)			495 (45)		115 (31.9)		380 (51.4)		<.001	
Dyslipidemia, n (%)			375 (34.1)		87 (24.2)		288 (38.9)		<.001	
Diabetes mellitus, n (%)			184 (16.7)		55 (15.3)		129 (17.4)		.37	
Chronic kidney disease, n (%)			17 (1.5)		3 (0.8)		14 (1.9)		.18	
Vision problems, n (%)			612 (55.6)		208 (57.8)		404 (54.6)		.32	
Wore glasses or contact lens, n (%)			399 (65.2)		144 (69.2)		255 (63.1)		.13	
Hearing problems, n (%)			120 (10.9)		15 (4.2)		105 (14.2)		<.001	
Used hearing aids, n (%)			4 (3.3)		0 (0)		4 (3.8)		.44	
Had experience using a smartphone or tablet, n (%)			873 (79.4)		332 (92.2)		541 (73.1)		<.001	
Had own smartphone, n (%)			843 (76.6)		317 (88.1)		526 (71.1)		<.001	
Had own tablet, n (%)			20 (1.8)		12 (3.3)		8 (1.1)		.009	
Had experience using the internet, n (%)			784 (71.3)		323 (89.7)		461 (62.3)		<.001	
Had experience in using mHealth^b^ apps, n (%)			439 (50.3)		205 (61.7)		234 (43.3)		<.001	
Intention to use mHealth apps, n (%)			537 (48.8)		220 (61.1)		317 (42.8)		<.001	

^a^THB 1=US $0.0296195.

^b^mHealth: mobile health.

### Dimensionality

According to the item analysis, we excluded 6 items from the physical function subscale (P2, P3, P4, P5, P7, and P8) due to a floor effect or ceiling effect of >80% (Table S3 in Multimedia Appendix 1). An EFA was conducted using PCA with 34 remaining items. The Bartlett test of sphericity obtained *P*<.001, indicating that the correlation matrix was not random [37]. The KMO statistic was 0.875, well above the minimum standard for conducting factor analysis [36]. Therefore, we determined that the input data were appropriate for EFA. Subsequently, the rotation of principal components was performed using both orthogonal rotation (varimax) and oblique rotation (promax) in an attempt to achieve a simple structure. Given the fact that an oblique rotation is generally recommended by measurement specialists to facilitate the emergence of factor intercorrelations [48-50], almost all social sciences measurements exhibit some degree of correlation [51]. In addition, the correlation matrix for the factors with oblique (promax) rotation indicated that the highest correlation was 0.445 (Table S3 in Multimedia Appendix 1); we thereby determined that the factors were correlated, and hence, oblique rotation was an appropriate approach. The results of parallel analysis (Table S4 in Multimedia Appendix 1) and PCA with or without oblique (promax) rotation all recommended the retention of 7 factors. According to the previous criteria, 2-item factors were identified, including factor 4 (PBR1 and PBR2) and factor 6 (S1 and S2). The internal consistency of seven factors demonstrated Cronbach α of 0.884 with 95% CI (0.875-0.894), which met acceptable thresholds. Within the context and theoretical framework of the STAM [24] and the UTAUT [11,21,26,52], social factors significantly influence behavioral intentions to use technology, particularly in the use of mHealth. Perceived barriers also play a role in determining intentions to use mHealth, as demonstrated by the aforementioned scoping review [31]. The inclusion of factor 4 and factor 6, which represented perceived barriers and social relationships, was considered appropriate. Based on the priori criteria and consensus of the panel experts, the EFA identified 22 candidate items (ATT1, ATT2, PU1, PU2, PU3, PEOU1, PEOU2, PB1, PB2, ANX1, ANX2, FC2, FC4, FC5, H1, H2, H5, C2, C3, C4, S1, and S2) with factor loadings greater than 0.4 that encompassed the 7 factors. The final EFA result is presented in Table 2.

**Table 2:** Exploratory factor analysis of the final 22-item Thai mobile health (mHealth) senior technology acceptance model (STAM).

Items	Factor loadings^a^								Communality value
	Factor 1	Factor 2	Factor 3	Factor 4	Factor 5	Factor 6	Factor 7		
ATT1	0.639^b^	0.096	0.057	0.027	0.040	0.057	0.060	0.824	
ATT2	0.631^b^	0.059	0.060	0.036	0.058	0.071	0.057	0.822	
PU1	0.899^b^	0.061	0.030	0.065	0.053	0.040	0.032	0.825	
PU2	0.921^b^	0.031	0.027	0.081	0.036	0.054	0.099	0.878	
PU3	0.919^b^	0.046	0.015	0.079	0.046	0.056	0.072	0.873	
PEOU1	0.473^b^	0.188	0.074	0.298	0.055	0.007	0.175	0.677	
PEOU2	0.513^b^	0.169	0.108	0.281	0.080	0.019	0.175	0.746	
PBR1	0.115	0.203	0.071	0.854^b^	0.025	0.000	0.038	0.795	
PBR2	0.141	0.200	0.061	0.855^b^	0.018	0.009	0.069	0.811	
PBR3	0.031	0.880^b^	0.025	0.120	–0.017	0.003	0.032	0.806	
PBR4	0.073	0.894^b^	0.012	0.109	–0.016	0.023	0.058	0.831	
ANX1	0.130	0.720^b^	0.061	0.183	0.015	0.019	0.044	0.734	
ANX2	0.107	0.643^b^	0.081	0.231	0.030	0.031	0.055	0.688	
FC2	0.324	0.088	0.032	0.103	0.034	0.054	0.579^b^	0.486	
FC4	0.380	0.171	0.147	0.237	0.064	–0.009	0.449^b^	0.606	
FC5	0.330	0.201	0.020	0.124	0.032	0.079	0.563^b^	0.504	
H1	0.074	–0.020	0.172	0.007	0.723^b^	0.100	0.017	0.573	
H2	0.092	–0.009	0.129	0.042	0.713^b^	0.097	0.009	0.556	
H5	0.127	–0.037	0.297	0.098	0.505^b^	0.112	0.072	0.498	
C1	0.009	0.040	0.614^b^	0.081	0.221	0.066	0.026	0.497	
C2	0.163	0.086	0.611^b^	0.164	0.177	0.117	0.073	0.582	
C3	0.019	0.054	0.693^b^	0.067	0.144	0.129	0.024	0.547	
C4	0.043	0.065	0.624^b^	0.068	0.106	0.237	0.024	0.497	
S1	0.126	0.040	0.226	0.021	0.147	0.669^b^	0.024	0.548	
S2	0.142	0.034	0.162	–0.012	0.116	0.678^b^	0.040	0.532	
% of variance^b^	26.41	15.99	12.37	11.77	10.76	7.32	6.83	—^c^	
Cumulative % of variance^b^	26.41	42.40	54.77	66.54	77.30	84.60	91.45	—	

^a^The extraction method was principal component analysis, with the rotation method by oblique, promax rotation.

^b^Items load on the assigned factor loadings >0.4 are highlighted.

^c^Not applicable.

### Construct Validity

From the EFA, the 22 items of the 7-factor Thai mHealth STAM explained 91.45% of the variance. The unidimensionality of each factor (subscale) and the overall models were assessed by analyzing modification indices in the CFA. Of the 7 factors from the EFA, the CFA of each factor (subscale) showed that only 5 factors, consisting of cognitive ability (C2, C3, C4), perceived barriers (PB1 and PB2), facilitating conditions (FC2, FC4, and FC5), self-reported health conditions (H1, H2, and H5), and social relationships (S1 and S2), showed satisfactory information criteria indices of the CFA, as presented Table 3. Factor 1, which included items from the attitude toward using (ATT1 and ATT2), perceived usefulness (PU1, PU2, and PU3), and perceived ease of use (PEOU1 and PEOU2), did not meet the CFA criteria due to over-factoring issues (Table S5 in Multimedia Appendix 1). Factor 2, combining perceived barriers (PB3 and PB4) and gerontechnology anxiety (ANX1 and ANX2), was unfit according to CFA criteria, with a low CFI (0.799), low TLI (0.698), and high RMSEA (0.306, 90% CI 0.293-0.319). Attempts to combine subscales also did not meet CFA criteria (Table S5 in Multimedia Appendix 1). However, when items were separated as in the original STAM, including attitude toward using (ATT1 and ATT2), perceived usefulness (PU1, PU2, and PU3), perceived ease of use (PEOU1 and PEOU2), and gerontechnology anxiety (ANX1 and ANX2), these separated factors showed a good fit with CFA criteria (Table S5 in Multimedia Appendix 1). Out of the 9 factors from the single latent factor analysis, 5 were 2-item factors. These were kept in the final CFA model because 3 factors (attitude toward using, perceived ease of use, and gerontechnology anxiety) were originally designed as 2-item factors, similar to the original STAM. The perceived barriers and social relationships were also retained because of their contextual relevance, as described above. Finally, the CFA confirmed 9-dimensional sets of 22 items with satisfactory fit indices, as shown in Table 3. The details of the CFAs of evaluated and reevaluated models are described in Table S4 in Multimedia Appendix 1.

A nonparametric IRT analysis also affirmed the unidimensionality, local independence, and monotonicity of the 22-item model with 8 factors (Table S6 in Multimedia Appendix 1). For the scalability, all 22 items of the Thai mHealth STAM had *H^s^* coefficients over 0.4, which indicates medium to strong scalability properties (Table S6 in Multimedia Appendix 1). The correlation among the final 22-item Thai mHealth STAM subscales ranged from 0.040 to 0.685 (Table S7 in Multimedia Appendix 1). The final 22-item Thai mHealth STAM questions, along with the English version and modeling indices, are described in Table 4.

Each item is scored on a 10-point Likert scale from 1 (very unsatisfied or strongly disagree) to 10 (very satisfied or strongly agree), with reverse scaling for perceived barriers and gerontechnology anxiety.

**Table 3:** Confirmatory factor analysis of the final Thai mobile health (mHealth) senior technology acceptance model (STAM).

Factor	Number of items	Threshold for acceptable fit						Model fit
		CFI^a^ (>0.90)	TLI^b^ (>0.90)	RMSEA^c^ (<0.10 [90% CI])	SRMR^d^ (<0.10)	*R*^2^ (>0.30)		
Attitude toward using	2 items (ATT1 and ATT2)	1.000	1.000	<0.001 (<0.001 to <0.001)	<0.001	All >0.30	Acceptable	
Perceived of benefits	3 items (PU1, PU2, and PU3)	1.000	1.000	<0.001 (<0.001 to <0.001	<0.001	All >0.30	Acceptable	
Perceived ease of use	2 items (PEOU1 and PEOU2)	1.000	1.000	<0.001 (<0.001 to <0.001)	<0.001	All >0.30	Acceptable	
Perceived of barriers	2 items (PB1 and PB2)	1.000	1.000	<0.001 (<0.001 to <0.001)	<0.001	All >0.30	Acceptable	
Gerontechnology anxiety	2 items (ANX1 and ANX2)	1.000	1.000	<0.001 (<0.001 to <0.001)	<0.001	All >0.30	Acceptable	
Facilitating conditions	3 items (FC2, FC4, and FC5)	1.000	1.000	<0.001 (<0.001 to <0.001)	<0.001	All >0.30	Acceptable	
Self-reported health conditions	3 items (H1, H2, and H5)	1.000	1.000	<0.001 (<0.001 to <0.001)	<0.001	All >0.30	Acceptable	
Cognitive ability	3 items (C2, C3, and C4)	1.000	1.000	<0.001 (<0.001 to <0.001)	<0.001	All >0.30	Acceptable	
Social relationships	2 items (S1 and S2)	1.000	1.000	<0.001 (<0.001 to <0.001)	<0.001	All >0.30	Acceptable	
Final Thai mHealth STAM 9-dimensional model	22 items	0.976	0.968	0.043 (0.039 to 0.047)	0.044	All >0.30	Acceptable	

^a^CFI: comparative-fit index.

^b^TLI: Tucker-Lewis Index.

^c^RMSEA: root mean square error of approximation.

^d^SRMR: standardized root mean squared residual.

**Table 4:** The final 22-item Thai mobile health (mHealth) senior technology acceptance model (STAM).

Items and questions				Mean (SD)		Ceiling, %		Floor, %		Skewness	Kurtosis		Standardized factor loading (95% CI)	*R* ^2^
**Attitude toward use**														
	ATT1	Using mobile health applications is a good idea.	8.18 (2.30)		48.45		2.50		–1.11		3.45	0.94 (0.92-0.95)		0.875
	ATT2	You like the idea of using mobile health applications.	8.02 (2.39)		45.85		3.11		0.34		2.25	0.93 (0.91-0.95)		0.862
**Perceived usefulness**														
	PU1	Using mobile health applications would enhance your effectiveness in life.	7.66 (2.59)		40.91		4.09		–0.92		2.93	0.91 (0.90-0.92)		0.831
	PU2	Using mobile health applications would make your life more convenient.	7.72 (2.61)		43.08		4.09		–0.95		2.93	0.94 (0.93-0.95)		0.897
	PU3	You would find mobile health applications useful in your life.	7.82 (2.62)		45.42		3.92		–1.03		3.03	0.94 (0.93-0.95)		0.891
**Perceived ease of use**														
	PEOU1	You would find mobile health applications are easy to use.	6.37 (3.18)		28		11.64		–0.34		1.76	0.83 (0.81-0.86)		0.693
	PEOU2	You could be skillful at using mobile health applications.	6.93 (3.06)		34		9		–0.62		2.10	0.89 (0.87-0.92)		0.803
**Gerontechnology anxiety**														
	ANX1	You feel apprehensive about using mobile health applications.	5.89 (3.16)		23.56		13.2		–0.09		1.70	0.89 (0.86-0.92)		0.799
	ANX2	You hesitate to use the technology for fear of making mistakes you cannot correct.	5.77 (3.11)		20.66		13.92		–0.38		1.75	0.95 (0.93-0.98)		0.918
**Perceived barriers**														
	PB1	You need to put in a lot of effort to use mobile health applications?	4.81 (3.14)		13.27		21.42		0.34		1.79	0.89 (0.86-0.92)		0.704
	PB2	You need to spend a lot of time to use mobile health applications?	5.00 (3.19)		14.78		20.35		0.26		1.70	0.96 (0.93-0.98)		0.819
**Facilitating conditions**														
	FC2	A specific person (or group) is available for assistance with difficulties using mobile health applications.	7.45 (3.09)		44.79		10.06		–0.96		2.57	0.63 (0.59-0.68)		0.407
	FC4	When you want or need to use mobile health applications, they are accessible to you.	7.20 (2.93)		36.92		7.83		–0.75		2.38	0.79 (0.76-0.83)		0.631
	FC5	Your family and friends think/support that you should use mobile health applications.	6.72 (3.33)		36.62		14.04		–0.55		1.83	0.68 (0.64-0.72)		0.461
**Self-reported health conditions**														
	H1	How are your general health conditions?	7.73 (1.75)		21.78		0.27		–0.52		2.79	0.80 (0.77-0.84)		0.651
	H2	How are your health conditions compared with the same-age groups?	7.91 (2.03)		33.69		0.18		–0.69		2.54	0.77 (0.73-0.81)		0.595
	H5	How well are you able to move around?	8.56 (1.96)		52.85		0.36		–1.37		4.24	0.61 (0.56-0.65)		0.370
**Cognitive ability**														
	C2	How satisﬁed are you with your ability to learn new information?	7.89 (2.25)		38.38		1.16		–0.98		3.27	0.65 (0.61-0.70)		0.432
	C3	How well are you able to concentrate?	8.75 (1.72)		54.23		0.18		–1.51		4.96	0.75 (0.71-0.79)		0.563
	C4	How satisﬁed are you with your ability to make decisions?	8.91 (1.58)		58.15		0.09		–1.48		4.64	0.76 (0.72-0.80)		0.578
**Social relationships**														
	S1	How satisfied are you with your personal relationships?	9.34 (1.29)		70.99		0.09		–2.36		8.95	0.84 (0.80-0.89)		0.712
	S2	How satisfied are you with the support you get from your friends and family?	9.39 (1.27)		74.01		0.09		–2.59		10.47	0.75 (0.71-0.80)		0.568
Overall (possible range 22-220)				164.16 (30.55)		—^a^		—		—	—		—	0.999

^a^Not applicable.

### Discriminant Validity

Considering the absence of a reference standard, it is theoretically reasonable that more participants with higher STAM scores will result in greater acceptance and adoption of technology. The discriminative indices, including sensitivity, specificity, and AUROC, were used to determine the cutoff scores for the proposed questionnaire, considering the intention to use mHealth from the external question. The 6 proposed sets of the final 22-item Thai mHealth STAM bands were classified into low, moderate, and high acceptance, as presented in Table 5. The set D of the possible banding was preferred as the optimal 22-item Thai mHealth STAM cutoff score based on the highest sensitivity of 89% (95% CI 86.1%-91.5%) and AUROC of 72.4% (95% CI 70%-74.8%). This finding also confirmed the discrimination performance of the 22-item Thai mHealth STAM in identifying persons with and without the intention to use mHealth. For set D, low, moderate, and high scores are defined as ≤151, 152-180, and ≥181, respectively. In addition, we conducted a subgroup analysis based on age groups: pre-older adults (aged 45-59 years) and older adults (aged 60 years and older). The result revealed that the set D banding had robust discriminant validity in older adults (AUROC 73%, 95% CI 70%-76%), but the discriminant validity decreased in the pre-older adult group (AUROC 67.7%, 95% CI 63.3%-71.9%). The discriminant validity of the 22-item Thai mHealth STAM by the subpopulation cohorts is shown in Table S8 in Multimedia Appendix 1.

**Table 5:** Proposed sets of the final 22-item Thai mobile health (mHealth) senior technology acceptance model (STAM) bands.

Possible bandings^a^			Discriminant validity (intention to use mHealth)			
Set and band		Score	aOR^b^ (95% CI)	Sensitivity (95% CI)	Specificity (95% CI)	AUROC^c^ (95% CI)
**Set A**						
	Low	≤121	Ref^d^	Ref	Ref	Ref
	Moderate	122-150	2.69^e^ (1.21-5.95)	98.5 (97.1-99.4)	16.0 (13.1-19.3)	57.3 (55.6-58.8)
	High	≥151	15.53^e^ (7.31-5.95)	89.0 (86.1-91.5)	53.5 (49.2-57.6)	71.2 (68.8-73.7)
**Set B**						
	Low	≤131	Ref	Ref	Ref	Ref
	Moderate	132- 160	4.37^e^ (2.42-7.88)	97.2 (95.4-98.4)	26.1 (22.5-29.9)	61.7 (59.7-63.6)
	High	≥161	15.53^e^ (8.75-27.52)	78.0 (74.3-81.5)	64.8 (60.7-68.8)	71.4 (68.8-74.1)
**Set C**						
	Low	≤141	Ref	Ref	Ref	Ref
	Moderate	142- 170	4.33^e^ (2.82-6.66)	93.7 (91.3-95.6)	40.9 (36.8-45.0)	67.3 (65.0-69.5)
	High	≥171	13.18^e^ (8.-20.26)	66.1 (61.9- 70.1)	75.8 (72.1-79.3)	71.0 (68.3-73.6)
**Set D**						
	Low	≤151	Ref	Ref	Ref	Ref
	Moderate	152-180	5.73^e^ (4.01–8.19)	89.0 (86.1-91.5)	55.8 (51.6-59.9)	72.4 (70.0-74.8)
	High	≥181	12.49^e^ (8.45-18.47)	49.9 (45.6-54.2)	84.7 (81.5–87.6)	67.3 (64.7-69.9)
**Set E**						
	Low	≤161	Ref	Ref	Ref	Ref
	Moderate	162-190	3.55^e^ (2.62-4.83)	76.7 (72.9-80.2)	65.5 (61.5-69.5)	71.1 (68.5-73.4)
	High	≥191	8.46^e^ (5.74-12.47)	37.4 (33.3-41.7)	90.9 (88.3-93.2)	64.2 (61.8-66.6)
**Set F**						
	Low	≤171	Ref	Ref	Ref	Ref
	Moderate	172-200	4.24^e^ (3.14-5.74)	65.2 (61.0-69.2)	77.1 (73.4-80.5)	71.1 (68.4-73.8)
	High	≥201	7.59^e^ (4.68-12.29)	21.8 (18.4-25.5)	95.6 (93.5-97.1)	58.7 (56.7-60.6)

^a^The final 22-item Thai mHealth STAM is highlighted.

^b^aOR: adjusted odds ratio.

^c^AUROC: area under receiver operating characteristic curve.

^d^Ref: reference.

^e^All reported aORs were statistically significant with *P* value<.05. aORs were estimated using a multivariable logistic regression with adjustment for age, gender, education levels (no, primary, secondary, and university education), income levels (low: <10,000 baht, moderate: 10,000-30,000 baht, and high: >30,000 bath), and living alone.

### Scale Reliability

Out of 1100 overall participants, the final 22-item Thai mHealth STAM demonstrated an excellent internal consistency in both the Cronbach α (0.88, 95% CI 0.87-0.89) and the McDonald ω coefficients (0.85, 95% CI 0.83-0.87), as shown in Table 6. By subpopulation, the Cronbach α and the McDonald ω coefficients were 0.88 (95% CI 0.86-0.90) and 0.84 (95% CI 0.81- 0.89) for adults aged 45-59 years and 0.88 (95% CI 0.86- 0.89) and 0.83 (95% CI 0.81-0.86) for older adults. All 22 items revealed the corrected item-total correlations ranging from 0.26 to 0.71, achieving a level of acceptance between 0.20 and 0.80 (Table 6).

**Table 6:** Reliability of the final 22-Item Thai mobile health (mHealth) senior technology acceptance model (STAM).

Items	n	Item-total correlations	Corrected item-total correlations	Average interitem correlation	Cronbach α	McDonald ω
ATT1	1158	0.652	0.605	1.682	0.877	0.879
ATT2	1156	0.652	0.603	1.675	0.877	0.879
PU1	1149	0.684	0.634	1.645	0.875	0.878
PU2	1149	0.720	0.673	1.631	0.874	0.876
PU3	1147	0.713	0.667	1.632	0.874	0.877
PEOU1	1143	0.721	0.664	1.585	0.873	0.878
PEOU2	1144	0.761	0.713	1.575	0.871	0.876
PB1	1130	0.514	0.431	1.682	0.881	0.885
PB2	1130	0.543	0.462	1.666	0.880	0.884
ANX1	1129	0.472	0.385	1.701	0.883	0.886
ANX2	1128	0.481	0.396	1.698	0.883	0.886
FC2	1123	0.546	0.469	1.671	0.880	0.884
FC4	1124	0.703	0.649	1.613	0.874	0.879
FC5	1125	0.586	0.507	1.640	0.879	0.883
H1	1125	0.316	0.263	1.805	0.885	0.888
H2	1125	0.342	0.281	1.790	0.884	0.887
H5	1124	0.401	0.344	1.775	0.883	0.886
C2	1123	0.522	0.465	1.725	0.880	0.883
C3	1123	0.340	0.288	1.800	0.884	0.887
C4	1123	0.366	0.320	1.799	0.883	0.886
S1	1117	0.343	0.305	1.814	0.884	0.886
S2	1116	0.329	0.291	1.817	0.884	0.887
Test scale, Cronbach α (95% CI)	—^a^	—	—	1.701	0.884 (0.875-0.894)	0.85 (0.83-0.87)

^a^Not applicable.

## Discussion

### Principal Findings

The study aimed to adapt and validate the STAM questionnaire for assessing mHealth technology acceptance among pre-older and older populations regarding the use of health support. The results confirmed the scale’s factor structure, supported an 8-factor model with 22 items, and showed good discriminant validity in predicting mHealth intention. The optimal version was a 22-item Thai mHealth STAM using the scoring cutoff (≥152). Subgroup analysis indicated no significant difference in discriminant validity between pre-older and older adults. The scale demonstrated strong internal consistency and stability, with reliability confirmed by Cronbach α and McDonald ω coefficients. This adapted 22-item version is more relevant for assessing mHealth intention among older adults and is suitable for public surveys and routine practice, which take less than 15 minutes to complete.

Our findings are consistent with the previous study conducted by the owner of the original STAM [53], which was subsequently developed into a brief form to save administration time and reduce the burden on respondents. The 14-item brief version of the STAM questionnaire consisted of a 4-factor structure: attitudinal beliefs, control beliefs, gerontechnology anxiety, and health. These findings are consistent with ours, reflecting the original STAM model constructs and the age-related health characteristics of older adults. We observed a decrease in discriminant validity within the pre-older adult group, indicating a need for additional factors to explain their behavioral intentions. For example, older adults with different genders, education levels, income, marital status, and ethnicity may have different intentions and purposes to use mHealth for their health [54].

### Strengths and Limitations

On the strength side, this is the first Thai version of the STAM questionnaire suitable for evaluating technology acceptance in Thai older adults. The 22-item Thai STAM version demonstrates structural balance, reliability, and validity in assessing technology acceptance among older individuals. The evaluation process is time-efficient. In addition, this tool can be used with both pre-older adults and older adults to prepare them for engaging with technology in their future lives. Furthermore, the evaluation takes into account the influence of Thai cultural norms on the adoption and acceptance of mHealth.

However, there are some limitations to consider. Although the psychometric properties of the 22-item Thai mHealth STAM are satisfied through transcultural adaptation in terms of validity and reliability in both the pre-older and older populations, this scale can be applied for use in a broad. However, our study participants may not be representative of the overall Thai pre-older and older populations, as almost all of the participants lived in the northern part of Thailand, particularly in Chiang Mai province. In order to address this concern, future studies, including those based on different regions of Thailand and other specific populations (eg, teenagers, vulnerable groups, minorities, and specific groups of patients) that could potentially derive advantages from mHealth usage, are recommended to expand the generalizability and usability of this scale. Finally, the 22-item Thai mHealth STAM was evaluated based on the board’s definition of mHealth. It is possible that the proposed questionnaire may not be compatible with all of the existing mHealth technologies due to the diverse range of mHealth technologies in health care. The patient’s choice may vary depending on several factors, such as health care providers, types of services, or the specific application. Hence, we suggest using this questionnaire to assess their acceptance and intention to use it in conjunction with the designated mHealth technology.

### Practical Implications of the 22-Item Thai mHealth STAM

The 22-item Thai mHealth STAM offers a practical assessment of patients’ acceptability—a crucial factor often overlooked, as evidenced by a recent systematic review of technology acceptability in health care, which revealed that only 10% (142/1219) of the reviewed studies examined patient acceptance [55]. This publicly available questionnaire has the potential to support health care professionals, policy makers, and developers in making informed decisions [56,57], particularly regarding the adoption and acceptance of mHealth within Thai cultural norms. This questionnaire can be incorporated into the research and development (R&D) processes of mHealth and used as a questionnaire to define the target population based on levels of acceptability, as well as ascertain the factors that encourage or hinder the adoption of their mHealth technologies [58,59]. This information is important for informing stakeholders and developers in advance of the mHealth R&D and implementation stages, which necessitate user data for resource allocation and planning in consideration of user requirements and experiences [60,61].

### Conclusion

The increasing number of older people, along with their growing adoption of technology, indicates that mHealth technologies might offer a new approach to enhancing the health of older adults with lower health care expenses. Although there are many advantages to using mHealth apps, it is important to consider their acceptance and intention to use them for health-related objectives. We proposed the 22-item Thai mHealth STAM as the questionnaire to evaluate the levels of acceptability and intention to use mHealth in the Thai community of pre-older and older adults. The 22-item Thai mHealth STAM has demonstrated satisfactory psychometric properties in terms of validity and reliability. As a result, it is now feasible to use this questionnaire in a public survey to support stakeholders in making informed decisions. Nevertheless, to improve generalizability and long-term use, further study is needed to investigate the various demographic groups with the specific mHealth interventions.

## Appendix

Multimedia Appendix 1 Supplementary tables.

## Abbreviations

aOR: adjusted odds ratio
AUROC: area under the receiver operating characteristic
CFA: confirmatory factor analysis
CFI: comparative fit index
COSMIN: Consensus-Based Standards for the Selection of Health Status Measurement Instruments
EFA: exploratory factor analysis
FC: facilitating condition
FITT: Fit between Individuals, Tasks, and Technology
Health-ITUES: Health Information Technology Usability Evaluation Scale
IRT: item response theory
ITC: International Test Commission
KMO: Kaiser-Meyer-Olkin
MAUQ: Mobile Health App Usability Questionnaire
mHealth: mobile health
PCA: principal component analysis
R&D: research and development
REDCap: Research Electronic Data Capture
RMSEA: root mean square error of approximation
SRMR: standardized root mean square residual
STAM: senior technology acceptance model
SUS: System Usability Scale
TAM: technology acceptance model
TLI: Tucker-Lewis Index
UTAUT: unified theory of acceptance and use of technology

## Data Availability

The data sets generated and analyzed during this study are not publicly available due to the institutional policies for protecting participant confidentiality but are available from the corresponding author on reasonable request.
