# Supplementary material for: Transcultural Adaptation, Validation, Psychometric Analysis, and Interpretation of the 22-Item Thai Senior Technology Acceptance Model for Mobile Health Apps: Cross-Sectional Study
Source: JMIR Aging. 2025 Mar 11;8:e60156. doi: 10.2196/60156 (PMC11937714; doi:10.2196/60156)
Supplement: Multimedia Appendix 1 [file aging_v8i1e60156_app1.docx]

**Table S1.** The full 40-items (10 dimensions) Thai mHealth STAM

| **Items** | **Questions (in Thai)** | **Scoring structure** | **Mean (SD); Median (range)** | **Ceiling effect (%); Floor effect (%)** | **Skewness; Kurtosis** | **Standardized factor loading (95%CI)** | **R^2^** |
| --- | --- | --- | --- | --- | --- | --- | --- |
| **Attitude towards using** | | | | | | | |
| ATT1 | Using mobile health applications is a good idea. (การใช้แอปพลิเคชันสุขภาพเคลื่อนที่เป็นเรื่องที่ดี) | Ten-point-Likert scale 1 (Strongly disagree) –2–3–4–5–6–7–8–9–10 (Strongly agree) | 8.18 (2.30)  9 (1-10) | 48.45%  2.50% | -1.11  3.45 | 0.93 (0.92 to 0.95) | 0.872 |
| ATT2 | You like the idea of using mobile health applications. (ท่านชอบแนวคิดของการใช้แอปพลิเคชันสุขภาพเคลื่อนที่) | Ten-point-Likert scale 1 (Strongly disagree) –2–3–4–5–6–7–8–9–10 (Strongly agree) | 8.02 (2.39)  9 (1-10) | 45.85%  3.11% | 0.34  2.25 | 0.93 (0.91 to 0.94) | 0.859 |
| **Perceived usefulness** | | | | | | | |
| PU1 | Using mobile health applications would enhance your effectiveness in life. (การใช้แอปพลิเคชันสุขภาพเคลื่อนที่สามารถส่งเสริมการดูแลสุขภาพของท่าน) | Ten-point-Likert scale 1 (Strongly disagree) –2–3–4–5–6–7–8–9–10 (Strongly agree) | 7.66 (2.59)  8 (1-10) | 40.91%  4.09% | -0.92  2.93 | 0.91 (0.90 to 0.92) | 0.828 |
| PU2 | Using mobile health applications would make your life more convenient. (การใช้แอปพลิเคชันสุขภาพเคลื่อนที่ทำให้ท่านสะดวกสบายในการดูแลสุขภาพของท่าน) | Ten-point-Likert scale 1 (Strongly disagree) –2–3–4–5–6–7–8–9–10 (Strongly agree) | 7.72 (2.61) | 43.08%  4.09% | -0.95  2.93 | 0.94 (0.93 to 0.95) | 0.896 |
| PU3 | You would find mobile health applications useful in your life. (ท่านรู้สึกว่าการใช้แอปพลิเคชันสุขภาพเคลื่อนที่เป็นประโยชน์ต่อท่าน) | Ten-point-Likert scale 1 (Strongly disagree) –2–3–4–5–6–7–8–9–10 (Strongly agree) | 7.82 (2.62)  9 (1-10) | 45.42%  3.92% | -1.03  3.03 | 0.94 (0.93 to 0.95) | 0.890 |
| **Perceived ease of use** | | | | | | | |
| PEOU1 | You would find mobile health applications are easy to use. (ท่านรู้สึกว่าการใช้แอปพลิเคชันสุขภาพเคลื่อนที่เป็นเรื่องง่าย) | Ten-point-Likert scale 1 (Strongly disagree) –2–3–4–5–6–7–8–9–10 (Strongly agree) | 6.37 (3.18)  7 (1-10) | 28.00% 11.64% | -0.34  1.76 | 0.84 (0.81 to 0.86) | 0.705 |
| PEOU2 | You could be skillful at using mobile health applications. (ท่านรู้สึกว่าท่านสามารถเรียนรู้การใช้แอปพลิเคชันสุขภาพเคลื่อนที่ได้) | Ten-point-Likert scale 1 (Strongly disagree) –2–3–4–5–6–7–8–9–10 (Strongly agree) | 6.93 (3.06)  8 (1-10) | 34.00%  9.00% | -0.62  2.10 | 0.89 (0.87 to 0.92) | 0.799 |
| **Gerontechnology anxiety** | | | | | | | |
| ANX1 | You feel apprehensive about using mobile health applications. (ท่านรู้สึกกังวลเกี่ยวกับการใช้แอปพลิเคชันสุขภาพเคลื่อนที่) | Ten-point-Likert scale (Reverse scale)  1 (Strongly agree) –2–3–4–5–6–7–8–9–10 (Strongly disagree) | 5.89 (3.16)  6 (1-10) | 23.56%  13.20% | -0.09  1.70 | 0.84 (0.80 to 0.88) | 0.723 |
| ANX2 | You hesitate to use the technology for fear of making mistakes you cannot correct. (ท่านรู้สึกลังเลที่จะใช้แอปพลิเคชันสุขภาพเคลื่อนที่ เนื่องจากกลัวว่าท่านจะไม่สามารถจัดการกับความผิดพลาดจากการใช้งานได้) | Ten-point-Likert scale (Reverse scale)  1 (Strongly agree) –2–3–4–5–6–7–8–9–10 (Strongly disagree) | 5.77 (3.11)  6 (1-10) | 20.66%  13.92% | -0.38  1.75 | 0.90 (0.86 to 0.95) | 0.797 |
| **Perceived barriers** | | | | | | | |
| PB1 | You need to put in a lot of effort to use mobile health applications? (ท่านต้องใช้ความพยายามเป็นอย่างมาก เพื่อใช้แอปพลิเคชันสุขภาพเคลื่อนที่) | Ten-point-Likert scale (Reverse scale)  1 (Strongly agree) –2–3–4–5–6–7–8–9–10 (Strongly disagree) | 4.81 (3.14)  4 (1-10) | 13.27%  21.42% | 0.34  1.79 | 0.91 (0.89 to 0.93) | 0.831 |
| PB2 | You need to spend a lot of time to use mobile health applications? (ท่านต้องใช้เวลาเป็นอย่างมาก เพื่อใช้แอปพลิเคชันสุขภาพเคลื่อนที่) | Ten-point-Likert scale (Reverse scale)  1 (Strongly agree) –2–3–4–5–6–7–8–9–10 (Strongly disagree) | 5.00 (3.19)  5 (1-10) | 14.78%  20.35% | 0.26  1.70 | 0.93 (0.91 to 0.95) | 0.866 |
| PB3 | You feel insecure about your personal data when using mobile health applications?  (ท่านรู้สึกว่าถึงความไม่ปลอดภัยต่อข้อมูลส่วนบุคคลของท่าน เมื่อใช้แอปพลิเคชันสุขภาพเคลื่อนที่) | Ten-point-Likert scale (Reverse scale)  1 (Strongly agree) –2–3–4–5–6–7–8–9–10 (Strongly disagree) | 5.38 (3.22)  6 (1-10) | 19.03%  18.32% | 0.09  1.66 | 0.37 (0.31 to 0.42) | 0.134 |
| PB4 | You distrust the providers of mobile health applications? (ท่านรู้สึกไม่ไว้วางใจผู้ให้บริการแอปพลิเคชันสุขภาพเคลื่อนที่) | Ten-point-Likert scale (Reverse scale)  1 (Strongly agree) –2–3–4–5–6–7–8–9–10 (Strongly disagree) | 5.62 (3.22)  6 (1-10) | 21.63%  16.40% | 0.001  1.67 | 0.38 (0.31 to 0.42) | 0.135 |
| **Facilitating conditions** | | | | | | | |
| FC1 | You have the knowledge necessary to use mobile health applications. (ท่านมีความรู้เพียงพอที่จะใช้แอปพลิเคชันสุขภาพเคลื่อนที่) | Ten-point-Likert scale 1 (Strongly disagree) –2–3–4–5–6–7–8–9–10 (Strongly agree) | 5.78 (2.94)  5 (1-10) | 17.62%  12.46% | -0.08  1.92 | 0.74 (0.70 to 0.77) | 0.542 |
| FC2 | A specific person (or group) is available for assistance with difficulties using mobile health applications. (ท่านมีคน หรือกลุ่มคนที่สามารถให้ความช่วยเหลือแก่ท่านได้ เกี่ยวกับการใช้แอปพลิเคชันสุขภาพเคลื่อนที่) | Ten-point-Likert scale 1 (Strongly disagree) –2–3–4–5–6–7–8–9–10 (Strongly agree) | 7.45 (3.09)  9 (1-10) | 44.79%  10.06% | -0.96  2.57 | 0.61 (0.57 to 0.66) | 0.374 |
| FC3 | Your financial status does not limit your activities in using mobile health applications.  (ท่านคิดว่าสถานภาพทางการเงินของท่าน ไม่เป็นข้อจำกัดในการใช้แอปพลิเคชันสุขภาพเคลื่อนที่) | Ten-point-Likert scale 1 (Strongly disagree) –2–3–4–5–6–7–8–9–10 (Strongly agree) | 6.29 (3.31)  7 (1-10) | 30.87%  15.04% | -0.30  1.68 | 0.29 (0.23 to 0.35) | 0.086 |
| FC4 | When you want or need to use mobile health applications, they are accessible to you. (ท่านคิดว่าท่านสามารถเข้าถึงแอปพลิเคชันสุขภาพเคลื่อนที่ หากท่านต้องการใช้บริการดังกล่าว) | Ten-point-Likert scale 1 (Strongly disagree) –2–3–4–5–6–7–8–9–10 (Strongly agree) | 7.20 (2.93)  8 (1-10) | 36.92%  7.83% | -0.75  2.38 | 0.78 (0.74 to 0.81) | 0.602 |
| FC5 | Your family and friends think/support that you should use mobile health applications. (ครอบครัวหรือเพื่อนของท่านสนับสนุนว่า ท่านควรจะใช้แอปพลิเคชันสุขภาพเคลื่อนที่) | Ten-point-Likert scale 1 (Strongly disagree) –2–3–4–5–6–7–8–9–10 (Strongly agree) | 6.72 (3.33)  8 (1-10) | 36.62%  14.04% | -0.55  1.83 | 0.64 (0.60 to 0.68) | 0.407 |
| **Self-reported health conditions** | | | | | | | |
| H1 | How are your general health conditions? (สุขภาพโดยรวมของท่านเป็นอย่างไร) | Ten-point-Likert scale 1 (Very poor) –2–3–4–5–6–7–8–9–10 (Very good) | 7.73 (1.75)  8 (1-10) | 21.78%  0.27% | -0.52  2.79 | 0.72 (0.68 to 0.76) | 0.519 |
| H2 | How is your health conditions compared with the same-age groups? (สุขภาพโดยรวมของท่านเป็นอย่างไร เมื่อเทียบกับบุคคลอื่นๆ ในวัยเดียวกัน) | Ten-point-Likert scale 1 (Very poor) –2–3–4–5–6–7–8–9–10 (Very good) | 7.91 (2.03)  8 (1-10) | 33.69%  0.18% | -0.69  2.54 | 0.70 (0.66 to 0.75) | 0.495 |
| H3 | How good is your hearing? (ความสามารถในการได้ยินของท่านเป็นอย่างไร) | Ten-point-Likert scale 1 (Very poor) –2–3–4–5–6–7–8–9–10 (Very good) | 9.22 (1.53)  10 (1-10) | 71.11%  0.36% | -2.46  9.74 | 0.47 (0.41 to 0.52) | 0.217 |
| H4 | How well can you see? (ความสามารถในการมองเห็นของท่านเป็นอย่างไร) | Ten-point-Likert scale 1 (Very poor) –2–3–4–5–6–7–8–9–10 (Very good) | 8.59 (1.75)  10 (1-10) | 50.13%  0.09% | -1.11  3.50 | 0.45 (0.40 to 0.51) | 0.207 |
| H5 | How well are you able to move around?  (ความสามารถในเคลื่อนไหวของท่านเป็นอย่างไร) | Ten-point-Likert scale 1 (Very poor) –2–3–4–5–6–7–8–9–10 (Very good) | 8.56 (1.96)  10 (1-10) | 52.85%  0.36% | -1.37  4.24 | 0.69 (0.64 to 0.73) | 0.473 |
| **Cognitive ability** | | | | | | | |
| C1 | How would you rate your memory? (ความจำของท่านเป็นอย่างไร) | Ten-point-Likert scale 1 (Very poor) –2–3–4–5–6–7–8–9–10 (Very good) | 8.26 (1.84)  8 (1-10) | 38.11%  0.45% | -1.08  3.98 | 0.67 (0.63 to 0.71) | 0.447 |
| C2 | How satisﬁed are you with your ability to learn new information? (ความสามารถในการเรียนรู้หรือรับข้อมูลใหม่ๆ ของท่านเป็นอย่างไร) | Ten-point-Likert scale 1 (Very unsatisfied) –2–3–4–5–6–7–8–9–10 (Very satisfied) | 7.89 (2.25)  8 (1-10) | 38.38%  1.16% | -0.98  3.27 | 0.72 (0.68 to 0.76) | 0.512 |
| C3 | How well are you able to concentrate?  (ความสามารถในการมีสมาธิจดจ่อของท่านเป็นอย่างไร) | Ten-point-Likert scale 1 (Very uneasy) –2–3–4–5–6–7–8–9–10 (Very easy) | 8.75 (1.72)  10 (1-10) | 54.23%  0.18% | -1.51  4.96 | 0.72 (0.68 to 0.76) | 0.520 |
| C4 | How satisﬁed are you with your ability to make decisions? (ความสามารถในการตัดสินใจของท่านเป็นอย่างไร) | Ten-point-Likert scale 1 (Very unsatisfied) –2–3–4–5–6–7–8–9–10 (Very satisfied) | 8.91 (1.58)  10 (1-10) | 58.15%  0.09% | -1.48  4.64 | 0.67 (0.64 to 0.72) | 0.460 |
| **Social relationships** | | | | | | | |
| S1 | How satisfied are you with your personal relationships? (ท่านพึงพอใจต่อความสัมพันธ์ส่วนตัวของท่านกับผู้อื่น) | Ten-point-Likert scale 1 (Very unsatisfied) –2–3–4–5–6–7–8–9–10 (Very satisfied) | 9.34 (1.29)  10 (1-10) | 70.99%  0.09% | -2.36  8.95 | 0.79 (0.75 to 0.83) | 0.624 |
| S2 | How satisfied are you with the support you get from your friends and family? (ท่านพึงพอใจต่อความช่วยเหลือที่ได้รับจากครอบครัวและเพื่อน) | Ten-point-Likert scale 1 (Very unsatisfied) –2–3–4–5–6–7–8–9–10 (Very satisfied) | 9.39 (1.27)  10. (1-10) | 74.01%  0.09% | -2.59  10.47 | 0.75 (0.71 to 0.79) | 0.563 |
| S3 | Do you participate in social or community activities? (ท่านได้มีส่วนร่วมกับกิจกรรมทางสังคมและชุมชน) | Ten-point-Likert scale 1 (Never) –2–3–4–5–6–7–8–9–10 (Always) | 8.63 (2.31)  10 (1-10) | 61.86%  3.04% | -1.88  5.78 | 0.39 (0.33 to 0.45) | 0.155 |
| **Attitude to ageing and life satisfaction** | | | | | | | |
| A1 | Do you feel that as you get older you are less useful? (ท่านคิดว่าหากท่านมีอายุมากขึ้น ท่านจะทำประโยชน์น้อยลง) | Ten-point-Likert scale (Reverse scale)  1 (Strongly agree)  –2–3–4–5–6–7–8–9–10 (Strongly disagree) | 4.81 (3.24)  4 (1-10) | 17.15%  21.75% | 0.42  1.77 | 0.18 (0.10 to 0.25) | 0.032 |
| A2 | How satisfied are you with your quality of life? (ท่านพึงพอใจต่อคุณภาพชีวิตของท่าน) | Ten-point-Likert scale 1 (Very unsatisfied) –2–3–4–5–6–7–8–9–10 (Very satisfied) | 8.97 (1.43)  10 (1-10) | 54.83%  0.09% | -1.62  5.98 | 0.58 (0.43 to 0.74) | 0.342 |
| **Physical function** | | | | | | | |
| P1 | Can you use the telephone? (ท่านสามารถใช้โทรศัพท์ได้) | Ten-point-Likert scale 1 (Without difficulty)  –2–3 (Slightly more difficulty) –4–5–6–  7 (Great difficulty)  –8–9–10 (Cannot do) | 8.51 (2.57)  10 (1-10) | 65.00%  5.47% | -1.77  5.07 | 0.39 (0.33 to 0.44) | 0.154 |
| **P2** | Can you go shopping for groceries? (ท่านสามารถจับจ่ายใช้สอยได้) | Ten-point-Likert scale 1 (Without difficulty)  –2–3 (Slightly more difficulty) –4–5–6–  7 (Great difficulty)  –8–9–10 (Cannot do) | 9.37 (1.64)  10 (1-10) | **80.60%**  1.28% | -3.29  14.40 | 0.70 (0.66 to 0.73) | 0.489 |
| **P3** | Can you prepare your own meals? (ท่านสามารถเตรียมอาหารได้) | Ten-point-Likert scale 1 (Without difficulty)  –2–3 (Slightly more difficulty) –4–5–6–  7 (Great difficulty)  –8–9–10 (Cannot do) | 9.43 (1.59)  10 (1-10) | **83.39%**  1.19% | -3.44  15.30 | 0.84 (0.82 to 0.86) | 0.703 |
| **P4** | Can you do housework independently? (ท่านสามารถทำงานบ้านได้) | Ten-point-Likert scale 1 (Without difficulty)  –2–3 (Slightly more difficulty) –4–5–6–  7 (Great difficulty)  –8–9–10 (Cannot do) | 9.42 (1.64)  10 (1-10) | **83.61%**  1.37% | -3.42  14.95 | 0.92 (0.91 to 0.94) | 0.856 |
| **P5** | Can you do your own laundry? (ท่านสามารถซัก รีด เสื้อผ้าได้) | Ten-point-Likert scale 1 (Without difficulty)  –2–3 (Slightly more difficulty) –4–5–6–  7 (Great difficulty)  –8–9–10 (Cannot do) | 9.32 (1.80)  10 (1-10) | **81.55%**  1.74% | -3.12  12.52 | 0.89 (0.88 to 0.91) | 0.798 |
| P6 | Can you travels on public transportation independently? (ท่านสามารถเดินทางไปยังสถานที่อื่น ที่อยู่ไกลกว่าระยะการเดินของท่านได้) | Ten-point-Likert scale 1 (Without difficulty)  –2–3 (Slightly more difficulty) –4–5–6–  7 (Great difficulty)  –8–9–10 (Cannot do) | 8.94 (2.21)  10 (1-10) | 73.27%  2.74% | -2.29  7.38 | 0.60 (0.56 to 0.64) | 0.361 |
| **P7** | Can you take medication by yourself?  (ท่านสามารถรับประทานยาเองได้) | Ten-point-Likert scale 1 (Without difficulty)  –2–3 (Slightly more difficulty) –4–5–6–  7 (Great difficulty)  –8–9–10 (Cannot do) | 9.74 (0.96)  10 (1-10) | **89.60%**  0.18% | -5.00  32.48 | 0.47 (0.42 to 0.52) | 0.223 |
| **P8** | Can you manage your own money?  (ท่านสามารถจัดการการเงินของท่านได้) | Ten-point-Likert scale 1 (Without difficulty)  –2–3 (Slightly more difficulty) –4–5–6–  7 (Great difficulty)  –8–9–10 (Cannot do) | 9.40 (1.50)  10 (1-10) | **80.64%**  0.46% | -3.00  12.38 | 0.63 (0.59 to 0.67) | 0.396 |
| **Overall** | | Possible range 40–400 | 310.40 (45.77)  313 (282-344) | – | – | – |  |

**Abbreviations**: CI, confidence interval; SD, standard deviation. **Noted**: Boldfaced items indicate findings of floor effect or ceiling effect of >80%.(P2-5 and P7-8)

**Table S2.** Participant characteristics of the study population in EFA and CFA cohorts

| **Characteristics** | **Total** | **EFA** | **CFA** | ***p*-value** |
| --- | --- | --- | --- | --- |
|  | N=1,100 | *n*=550 | *n*=550 |  |
| Age (year), mean (SD) | 62.3 (8.8) | 62.3 (8.6) | 62.2 (9.0) | 0.930 |
| Male | 324 (29.5%) | 99 (27.5%) | 171 (31.1%) | 0.230 |
| Marital status |  |  |  | 0.220 |
| Single | 96 (8.7%) | 40 (7.3%) | 56 (10.2%) |  |
| Married | 747 (67.9%) | 382 (69.5%) | 365 (66.4%) |  |
| Separate/Divorce/widowed | 257 (23.4%) | 128 (23.3%) | 129 (23.5%) |  |
| Education levels |  |  |  | 0.430 |
| No education | 18 (1.6%) | 10 (1.8%) | 8 (1.5%) |  |
| Primary school | 725 (65.9%) | 365 (66.4%) | 360 (65.5%) |  |
| Secondary school | 97 (8.8%) | 47 (8.5%) | 50 (9.1%) |  |
| High school and vocational training | 162 (14.7%) | 80 (14.5%) | 82 (14.9%) |  |
| Pre-university | 11 (1.0%) | 2 (0.4%) | 9 (1.6%) |  |
| Brachelor’s degree | 79 (7.2%) | 43 (7.8%) | 36 (6.5%) |  |
| Master’s degree | 8 (0.7%) | 3 (0.5%) | 5 (0.9%) |  |
| Income |  |  |  | 0.660 |
| < 10,000 Baht | 948 (86.2%) | 469 (85.3%) | 479 (87.1%) |  |
| 10,001-30,000 Baht | 138 (12.5%) | 74 (13.5%) | 64 (11.6%) |  |
| > 30,001 Baht | 14 (1.3%) | 7 (1.3%) | 7 (1.3%) |  |
| Living status |  |  |  | 0.370 |
| Alone | 108 (9.8%) | 49 (8.9%) | 59 (10.7%) |  |
| With family | 988 (89.8%) | 498 (90.5%) | 490 (89.1%) |  |
| With others | 4 (0.4%) | 3 (0.5%) | 1 (0.2%) |  |
| Living area |  |  |  | 0.780 |
| Urban | 220 (20.0%) | 114 (20.7%) | 106 (19.3%) |  |
| Sub-urban | 377 (34.3%) | 184 (33.5%) | 193 (35.1%) |  |
| Rural | 503 (45.7%) | 252 (45.8%) | 251 (45.6%) |  |
| Had any underlying disease | 726 (66.0%) | 374 (68.0%) | 352 (64.0%) | 0.160 |
| Hypertension | 495 (45.0%) | 264 (48.0%) | 231 (42.0%) | 0.046 |
| Dyslipidemia | 375 (34.1%) | 203 (36.9%) | 172 (31.3%) | 0.049 |
| Diabetes Mellitus | 184 (16.7%) | 96 (17.5%) | 88 (16.0%) | 0.520 |
| Chronic kidney disease | 17 (1.5%) | 6 (1.1%) | 11 (2.0%) | 0.220 |
| Vision problems | 612 (55.6%) | 286 (52.0%) | 326 (59.3%) | 0.015 |
| Wore glasses or contact lens | 399 (65.2%) | 187 (65.4%) | 212 (65.0%) | 0.930 |
| Hearing problems | 120 (10.9%) | 56 (10.2%) | 64 (11.6%) | 0.440 |
| Used hearing aids | 4 (3.3%) | 2 (3.6%) | 2 (3.1%) | 0.890 |
| Had an experience on using smart phone or tablet | 873 (79.4%) | 436 (79.3%) | 437 (79.5%) | 0.940 |
| Had own’s smart phone | 843 (76.6%) | 423 (76.9%) | 420 (76.4%) | 0.830 |
| Had own’s tablet | 20 (1.8%) | 7 (1.3%) | 13 (2.4%) | 0.180 |
| Had an experience on using internet | 784 (71.3%) | 390 (70.9%) | 394 (71.6%) | 0.790 |
| Had an experience on using mHealth applications | 439 (50.3%) | 221 (50.7%) | 218 (49.9%) | 0.810 |
| Intention to use mHealth applications | 537 (48.8%) | 267 (48.5%) | 270 (49.1%) | 0.860 |

**Abbreviations**: CFA, confirmatory factor analysis; EFA, exploratory factor analysis; mHealth, mobile health applications; SD, standard deviation; STAM, senior technology acceptance model.

**Table S3.** Exploratory factor analysis of the final 22-items Thai mHealth STAM

| **Items** | **Factor Loadings†** | | | | | | | **Communality Value** |
| --- | --- | --- | --- | --- | --- | --- | --- | --- |
|  | **Factor1** | **Factor2** | **Factor3** | **Factor4** | **Factor5** | **Factor6** | **Factor7** |  |
| ATT1 | **0.639** | 0.096 | 0.057 | 0.027 | 0.040 | 0.057 | 0.060 | 0.824 |
| ATT2 | **0.631** | 0.059 | 0.060 | 0.036 | 0.058 | 0.071 | 0.057 | 0.822 |
| PU1 | **0.899** | 0.061 | 0.030 | 0.065 | 0.053 | 0.040 | 0.032 | 0.825 |
| PU2 | **0.921** | 0.031 | 0.027 | 0.081 | 0.036 | 0.054 | 0.099 | 0.878 |
| PU3 | **0.919** | 0.046 | 0.015 | 0.079 | 0.046 | 0.056 | 0.072 | 0.873 |
| PEOU1 | **0.473** | 0.188 | 0.074 | 0.298 | 0.055 | 0.007 | 0.175 | 0.677 |
| PEOU2 | **0.513** | 0.169 | 0.108 | 0.281 | 0.080 | 0.019 | 0.175 | 0.746 |
| PBR1 | 0.115 | 0.203 | 0.071 | **0.854** | 0.025 | 0.000 | 0.038 | 0.795 |
| PBR2 | 0.141 | 0.200 | 0.061 | **0.855** | 0.018 | 0.009 | 0.069 | 0.811 |
| PBR3 | 0.031 | **0.880** | 0.025 | 0.120 | -0.017 | 0.003 | 0.032 | 0.806 |
| PBR4 | 0.073 | **0.894** | 0.012 | 0.109 | -0.016 | 0.023 | 0.058 | 0.831 |
| ANX1 | 0.130 | **0.720** | 0.061 | 0.183 | 0.015 | 0.019 | 0.044 | 0.734 |
| ANX2 | 0.107 | **0.643** | 0.081 | 0.231 | 0.030 | 0.031 | 0.055 | 0.688 |
| FC2 | 0.324 | 0.088 | 0.032 | 0.103 | 0.034 | 0.054 | **0.579** | 0.486 |
| FC4 | 0.380 | 0.171 | 0.147 | 0.237 | 0.064 | -0.009 | **0.449** | 0.606 |
| FC5 | 0.330 | 0.201 | 0.020 | 0.124 | 0.032 | 0.079 | **0.563** | 0.504 |
| H1 | 0.074 | -0.020 | 0.172 | 0.007 | **0.723** | 0.100 | 0.017 | 0.573 |
| H2 | 0.092 | -0.009 | 0.129 | 0.042 | **0.713** | 0.097 | 0.009 | 0.556 |
| H5 | 0.127 | -0.037 | 0.297 | 0.098 | **0.505** | 0.112 | 0.072 | 0.498 |
| C1 | 0.009 | 0.040 | **0.614** | 0.081 | 0.221 | 0.066 | 0.026 | 0.497 |
| C2 | 0.163 | 0.086 | **0.611** | 0.164 | 0.177 | 0.117 | 0.073 | 0.582 |
| C3 | 0.019 | 0.054 | **0.693** | 0.067 | 0.144 | 0.129 | 0.024 | 0.547 |
| C4 | 0.043 | 0.065 | **0.624** | 0.068 | 0.106 | 0.237 | 0.024 | 0.497 |
| S1 | 0.126 | 0.040 | 0.226 | 0.021 | 0.147 | **0.669** | 0.024 | 0.548 |
| S2 | 0.142 | 0.034 | 0.162 | -0.012 | 0.116 | **0.678** | 0.040 | 0.532 |
| **% of variance** | 26.41 | 15.99 | 12.37 | 11.77 | 10.76 | 7.32 | 6.83 |  |
| **Cumulative**  **% of variance** | 26.41 | 42.40 | 54.77 | 66.54 | 77.30 | 84.60 | 91.45 |  |

†The extraction method was principal component analysis, with the rotation method by oblique, promax rotation. Items load on the assigned factor loadings >0.4 are highlighted.

**Table S4.** Confirmatory analysis of Thai mHealth STAM

| **Factor** | **Number of items** | **Threshold for acceptable fit** | | | | | **Model fit** |
| --- | --- | --- | --- | --- | --- | --- | --- |
|  |  | **CFI (>0.90)** | **TLI (>0.90)** | **RMSEA (<0.10 [90%CI])** | **SRMR (<0.10)** | **R-squared**  **(>0.30)** |  |
| Attitude towards using | 2 items (ATT1, ATT2) | 1.000 | 1.000 | <0.001  (<0.001 − <0.001) | <0.001 | All >0.30 | Acceptable/good |
| Perceived of benefits | 3 items (PU1, PU2, PU3) | 1.000 | 1.000 | <0.001  (<0.001 − <0.001 | <0.001 | All >0.30 | Acceptable/good |
| Attitude towards using + Perceived of benefits | 5 items (ATT1, ATT2, PU1, PU2, PU3) | 0.843 | 0.685 | 0.405  (0.383 − 0.427) | 0.095 | All >0.30 | Unacceptable |
| Perceived ease of use | 2 items (PEOU1, PEOU2) | 1.000 | 1.000 | <0.001  (<0.001 − <0.001) | <0.001 | All >0.30 | Acceptable/good |
| Attitude towards using + Perceived of benefits + Perceived ease of use | 7 items (ATT1, ATT2, PU1, PU2, PU3, PEOU1, PEOU2) | 0.799 | 0.698 | 0.306  (0.293 – 0.319) | 0.102 | All >0.30 | Unacceptable |
| Perceived of barriers | 4 items (PB1, PB2, PB3, PB4) | 0.555 | -0.336 | 0.787  (0.753 − 0.822) | 0.236 | PB3 = 0.12  PB4 = 0.11 Otherwise >0.30 | Unacceptable |
|  | 2 items (PB1, PB2) | 1.000 | 1.000 | <0.001  (<0.001 − <0.001) | <0.001 | All >0.30 | Acceptable/good |
| Gerontechnology anxiety | 2 items (ANX1, ANX2) | 1.000 | 1.000 | <0.001  (<0.001 − <0.001) | <0.001 | All >0.30 | Acceptable/good |
| Perceived of barriers + Gerontechnology anxiety | 4 items (PB3, PB4, ANX1, ANX2) | 0.864 | 0.592 | 0.437  (0.402 − 0.472) | 0.091 | All >0.30 | Unacceptable |
| Facilitating conditions | 5 items (FC1, FC2, FC3, FC4, FC5) | 0.953 | 0.905 | 0.109  (0.088 − 0.132) | 0.036 | FC3 = 0.10 Otherwise >0.30 | Unacceptable |
|  | 3 items (FC2, FC4, FC5) | 1.000 | 1.000 | <0.001  (<0.001 − <0.001) | <0.001 | All >0.30 | Acceptable/good |
| Self-reported health conditions | 5 items (H1, H2, H3, H4, H5) | 0.881 | 0.763 | <0.175  (<0.154 − <0.001) | 0.074 | H3 = 0.20  H4 = 0.20 Otherwise >0.30 | Unacceptable |
|  | 3 items (H1, H2, H5) | 1.000 | 1.000 | <0.001  (<0.001 − <0.001) | <0.001 | All >0.30 | Acceptable/good |
| Cognitive ability | 4 items (C1, C2, C3, C4) | 0.955 | 0.864 | 0.169  (0.135 – 0.205) | 0.040 | All >0.30 | Unacceptable |
|  | 3 items (C2, C3, C4) | 1.000 | 1.000 | <0.001  (<0.001 − <0.001) | <0.001 | All >0.30 | Acceptable/good |
| Social relationships | 3 items (S1, S2, S3) | 1.000 | 1.000 | <0.001  (<0.001 − <0.001) | <0.001 | S3 = 0.13 Otherwise >0.30 | Unacceptable |
|  | 2 items (S1, S2) | 1.000 | 1.000 | <0.001  (<0.001 − <0.001) | <0.001 | All >0.30 | Acceptable/good |
| Attitude to ageing and life satisfaction | 2 items (A1, A2) | 1.000 | 1.000 | <0.001  (<0.001 − <0.001) | <0.001 | A1 = 1.00  A2 = 0.01 | Unacceptable |
| Physical function | 8 items (P1, P2, P3, P4, P5, P6, P7, P8) | 0.881 | 0.833 | 0.165  (0.154 – 0.176) | 0.076 | P1 = 0.14  P7 = 0.21  Otherwise >0.30 | Unacceptable |
|  | 2 items (P1, P6) | 1.000 | 1.000 | <0.001  (<0.001 − <0.001) | <0.001 | P1 = 1.00  P6 = 0.13 | Unacceptable |
| Full Thai mHealth STAM  10-dimensional model | 40 Items (ATT1, ATT2, PU1, PU2, PU3, PEOU1, PEOU2, PB1, PB2, PB3, PB4, ANX1, ANX2, FC1, FC2, FC3, FC4, FC5, H1, H2, H3, H4, H5, C1, C2, C3, C4, S1, S2, S3, A1, A2, P1, P2, P3, P4, P5, P6, P7, P8) | 0.837 | 0.815 | 0.074  (0.072 − 0.076) | 0.075 | PB3 = 0.13  PB4 = 0.13  FC3 =0.09  H3 = 0.22  H4 = 0.20  S3 = 0.15  A1 = 0.03  P1 = 0.15  P7 = 0.22  Otherwise >0.30 | Unacceptable |
| Final Thai mHealth STAM  8-dimensional model | 22 Items (ATT1, ATT2, PU1, PU2, PU3, PEOU1, PEOU2, PB1, PB2, ANX1, ANX2, FC2, FC4, FC5, H1, H2, H5, C2, C3, C4, S1, S2) | 0.976 | 0.968 | 0.043  (0.039 – 0.047) | 0.044 | All >0.30 | Acceptable/good |

**Abbreviations**: CFI, comparative-fit index; CI, confidence interval; mHealth, mobile health applications; RMSEA, root mean square error of approximation; SRMR, standardized root mean squared residual; STAM, senior technology acceptance model; TLI, Tucker-Lewis index.

**Table S5.** Results of nonparametric Item Response Theory (IRT) analysis of the final 22-items Thai mHealth STAM (N=1,100)

| **Items** | **Loevinger’s H**  **Coefficients (*H^s^*)†** | **Z-statistics** | ***p*-value** | **Monotonicity Assumption**  **(Criterion <80)** |
| --- | --- | --- | --- | --- |
| **Attitude towards using** | | | | |
| ATT1 | 0.88 | 29.61 | <0.001 | –18 |
| ATT2 | 0.88 | 29.61 | <0.001 | –19 |
| **Perceived usefulness** | | | | |
| PU1 | 0.87 | 41.20 | <0.001 | 1 |
| PU2 | 0.88 | 41.91 | <0.001 | 35 |
| PU3 | 0.88 | 41.92 | <0.001 | 35 |
| **Perceived ease of use** | | | | |
| PEOU1 | 0.77 | 25.32 | <0.001 | –7 |
| PEOU2 | 0.77 | 25.32 | <0.001 | 19 |
| **Gerontechnology anxiety** | | | | |
| ANX1 | 0.86 | 28.81 | <0.001 | –10 |
| ANX2 | 0.86 | 28.81 | <0.001 | –10 |
| **Perceived barriers** | | | | |
| PB1 | 0.76 | 25.34 | <0.001 | 32 |
| PB2 | 0.76 | 25.34 | <0.001 | 61 |
| **Facilitating conditions** | | | | |
| FC2 | 0.53 | 24.23 | <0.001 | -11 |
| FC4 | 0.50 | 23.21 | <0.001 | -10 |
| FC5 | 0.55 | 25.15 | <0.001 | -12 |
| **Self-reported health conditions** | | | | |
| H1 | 0.60 | 26.64 | <0.001 | 16 |
| H2 | 0.57 | 25.64 | <0.001 | 47 |
| H5 | 0.50 | 21.81 | <0.001 | 45 |
| **Cognitive ability** | | | | |
| C2 | 0.50 | 22.56 | <0.001 | 23 |
| C3 | 0.55 | 25.07 | <0.001 | 49 |
| C4 | 0.54 | 24.52 | <0.001 | 66 |
| **Social relationships** | | | | |
| S1 | 0.65 | 21.35 | <0.001 | 46 |
| S2 | 0.65 | 21.35 | <0.001 | –17 |

**Abbreviations:** mHealth, mobile health applications; STAM, senior technology acceptance model.

†Loevinger’s H Coefficients indicates that, if *H^s^* <0.3, the scale has poor scalability properties; 0.3 ≤ *H^s^* <0.4, the scale is weak; 0.4 ≤ *H^s^* <0.5, the scale is medium; and *H^s^* ≥0.5, the scale is strong.

**Table S6.** Correlation among the final 22-Item Thai mHealth STAM subscales (N=1,100)

| **Subscales** | **Correlation (95% CI)** | | | | | | | |
| --- | --- | --- | --- | --- | --- | --- | --- | --- |
|  | Attitude towards using | Perceived of benefits | Perceived ease of use | Perceived of barriers | Gerontechnology anxiety | Facilitating conditions | Self-reported health conditions | Cognitive ability |
| Perceived of benefits | 0.685  (0.652 – 0.715) |  |  |  |  |  |  |  |
| Perceived ease of use | 0.505  (0.460 – 0.548) | 0.594  (0.554 – 0.631) |  |  |  |  |  |  |
| Perceived of barriers | 0.149  (0.090 – 0.206) | 0.211  (0.154 – 0.267) | 0.443  (0.395 – 0.490) |  |  |  |  |  |
| Gerontechnology anxiety | 0.066  (0.007 – 0.125) | 0.093  (0.034 – 0.151) | 0.069  (0.010 – 0.128) | 0.080  (0.021 – 0.138) |  |  |  |  |
| Facilitating conditions | 0.425  (0.375 – 0.472) | 0.499  (0.454 – 0.542) | 0.572  (0.531 – 0.610) | 0.307  (0.252 – 0.360) | 0.049  (–0.010 – 0.108) |  |  |  |
| Self-reported health conditions | 0.181  (0.123 – 0.237) | 0.185  (0.127 – 0.242) | 0.206  (0.149 – 0.262) | 0.101  (0.042 – 0.159) | 0.087  (0.028 – 0.146) | 0.180  (0.122 – 0.236) |  |  |
| Cognitive  ability | 0.181  (0.123 – 0.237) | 0.153  (0.094 – 0.210) | 0.267  (0.211 – 0.321) | 0.196  (0.139 – 0.252) | 0.085  (0.026 – 0.143) | 0.226  (0.169 – 0.281) | 0.447  (0.398 – 0.493) |  |
| Social relationships | 0.213  (0.156 – 0.269) | 0.204  (0.147 – 0.260) | 0.157  (0.099 – 0.215) | 0.046  (–0.013 – 0.105) | 0.040  (–0.019 – 0.099) | 0.183  (0.125 – 0.240) | 0.330  (0.277 – 0.382) | 0.412  (0.362 – 0.460) |

**Abbreviations**: CI, confidence interval; mHealth, mobile health applications; STAM, senior technology acceptance model.

**Table S7.** Item-total correlations and corrected Item-test correlations of the final 22-Item Thai mHealth STAM

| **Items** | ***n*** | **Sign** | **Item-total**  **correlations** | **Corrected**  **Item-total**  **correlations** | **Average**  **interitem**  **correlation** | **Cronbach's alpha** |
| --- | --- | --- | --- | --- | --- | --- |
| ATT1 | 1158 | + | 0.652 | 0.605 | 1.682 | 0.877 |
| ATT2 | 1156 | + | 0.652 | 0.603 | 1.675 | 0.877 |
| PU1 | 1149 | + | 0.684 | 0.634 | 1.645 | 0.875 |
| PU2 | 1149 | + | 0.720 | 0.673 | 1.631 | 0.874 |
| PU3 | 1147 | + | 0.713 | 0.667 | 1.632 | 0.874 |
| PEOU1 | 1143 | + | 0.721 | 0.664 | 1.585 | 0.873 |
| PEOU2 | 1144 | + | 0.761 | 0.713 | 1.575 | 0.871 |
| PB1 | 1130 | + | 0.514 | 0.431 | 1.682 | 0.881 |
| PB2 | 1130 | + | 0.543 | 0.462 | 1.666 | 0.880 |
| ANX1 | 1129 | + | 0.472 | 0.385 | 1.701 | 0.883 |
| ANX2 | 1128 | + | 0.481 | 0.396 | 1.698 | 0.883 |
| FC2 | 1123 | + | 0.546 | 0.469 | 1.671 | 0.880 |
| FC4 | 1124 | + | 0.703 | 0.649 | 1.613 | 0.874 |
| FC5 | 1125 | + | 0.586 | 0.507 | 1.640 | 0.879 |
| H1 | 1125 | + | 0.316 | 0.263 | 1.805 | 0.885 |
| H2 | 1125 | + | 0.342 | 0.281 | 1.790 | 0.884 |
| H5 | 1124 | + | 0.401 | 0.344 | 1.775 | 0.883 |
| C2 | 1123 | + | 0.522 | 0.465 | 1.725 | 0.880 |
| C3 | 1123 | + | 0.340 | 0.288 | 1.800 | 0.884 |
| C4 | 1123 | + | 0.366 | 0.320 | 1.799 | 0.883 |
| S1 | 1117 | + | 0.343 | 0.305 | 1.814 | 0.884 |
| S2 | 1116 | + | 0.329 | 0.291 | 1.817 | 0.884 |
| **Test scale** |  |  |  |  | **1.701** | **0.884**  **95% CI (0.875 – 0.894)** |

**Abbreviations**: CI, confidence interval; mHealth, mobile health applications; STAM, senior technology acceptance model.

**Table S8.** Discriminant validity of the 22-item Thai mHealth STAM by the subpopulation cohorts.

| **Possible bandings†** | | | **Discriminant validity (Intention to use mHealth)** | | |
| --- | --- | --- | --- | --- | --- |
|  | **Bands** | **Scores** | **Overall**  **(N=1100)** | **Pre-older**  **45-59 years**  **(*n*=360)** | **Older adult**  **60 years**  **(*n*=740)** |
|  |  |  | **AuROC (95% CI)** | **AuROC (95% CI)** | **AuROC (95% CI)** |
| Set A | Low | ≤121 |  | Ref. |  |
|  | Moderate | 122 – 150 | 57.3  (55.6 – 58.8) | 53.7  (51.2 – 56.3) | 58.5*  (56.5 – 60.4) |
|  | High | ≥151 | 71.2  (68.8 – 73.7) | 66.9  (62.5 – 71.2) | 71.7  (68.6 – 74.7) |
| Set B | Low | ≤131 |  | Ref. |  |
|  | Moderate | 132 – 160 | 61.7  (59.7 – 63.6) | 56.0  (52.9 – 59.1) | 63.4*  (61.0 – 65.8) |
|  | High | ≥161 | 71.4  (68.8 – 74.1) | 68.1  (63.3 – 72.8) | 70.9  (67.6 – 74.2) |
| Set C | Low | ≤141 |  | Ref. |  |
|  | Moderate | 142 – 170 | 67.3  (65.0 – 69.5) | 61.3  (57.4 – 65.1) | 68.7*  (65.8 – 71.5) |
|  | High | ≥171 | 71.0  (68.3 – 73.6) | 71.3  (66.4 – 76.1) | 69.0  (65.6 – 72.3) |
| **Set D** | Low | ≤151 |  | Ref. |  |
|  | **Moderate** | **152 – 180** | **72.4**  **(70.0 – 74.8)** | **67.6**  **(63.3 – 71.9)** | **73.0**  **(70.0 – 76.0)** |
|  | High | ≥181 | 67.3  (64.7 – 69.9) | 69.0  (64.2 – 73.8) | 64.5  (61.4 – 67.7) |
| Set E | Low | ≤161 |  | Ref. |  |
|  | Moderate | 162 – 190 | 71.1  (68.5 – 73.4) | 67.9  (63.2 – 72.7) | 70.5  (67.2 – 73.8) |
|  | High | ≥191 | 64.2  (61.8 – 66.6) | 66.9  (62.4 – 71.4) | 61.1  (58.3 – 63.9) |
| Set F | Low | ≤171 |  | Ref. |  |
|  | Moderate | 172 – 200 | 71.1  (68.4 – 73.8) | 70.8  (65.9 – 75.6) | 69.3  (65.9 – 72.6) |
|  | High | ≥201 | 58.7  (56.7 – 60.6) | 60.8  (56.9 – 64.7) | 56.3  (54.2 – 58.4) |

**Abbreviations**: CI, confidence interval; mHealth, mobile health applications; STAM, senior technology acceptance model.

* indicated a statistical difference (*p* <0.050) of AuROC between two subpopulations.
